# Supplementary material for: Mitochondrial Respiration Changes in R6/2 Huntington’s Disease Model Mice during Aging in a Brain Region Specific Manner
Source: Int J Mol Sci. 2020 Jul 30;21(15):5412. doi: 10.3390/ijms21155412 (PMC7432063; doi:10.3390/ijms21155412)
Supplement: Supplementary file 1 [file ijms-21-05412-s001.pdf]

International Journal of *Molecular Sciences*

MDPI

Supplementary material

Table S1 – Numerical overview on results presented in main figures 1 and 2. Mean ± SEM are indicated.

		state	4w WT	4w R6/2	10-11w WT	10-11w R6/2
**Striatum**	O_2 pmol/(s*mg)	**N_L**	13,59 ± 1,62	13,47 ± 1,86	13,26 ± 0,97	10,31 ± 0,34
		**N_P**	43,32 ± 3,69	38,21 ± 6,77	65,18 ± 0,81	65,14 ± 7,14
		**NS_P**	79,71 ± 5,80	86,70 ± 5,02	111,13 ± 3,30	107,20 ± 4,94
		**NS_E**	117,47 ± 8,65	125,44 ± 5,60	154,81 ± 5,19	147,61 ± 4,56
		**S_E**	49,90 ± 3,23	56,59 ± 2,24	59,86 ± 2,08	58,97 ± 0,90
		**CIV**	133,66 ± 6,47	142,66 ± 11,67	167,75 ± 9,77	161,57 ± 15,86
	FCR	**N_L**	0,12 ± 0,01	0,11 ± 0,01	0,09 ± 0,01	0,07 ± 0,00
		**N_P**	0,37 ± 0,02	0,30 ± 0,04	0,42 ± 0,01	0,44 ± 0,04
		**NS_P**	0,68 ± 0,01	0,69 ± 0,01	0,72 ± 0,01	0,73 ± 0,02
		**S_E**	0,43 ± 0,01	0,45 ± 0,00	0,39 ± 0,01	0,40 ± 0,02
		**CIV**	1,15 ± 0,05	1,14 ± 0,05	1,08 ± 0,06	1,10 ± 0,13
**Hippocampus**	O_2 pmol/(s*mg)	**N_L**	13,23 ± 1,62	11,78 ± 1,02	12,40 ± 0,63	9,92 ± 0,78
		**N_P**	48,6 ± 8,55	35,18 ± 5,25	55,52 ± 3,18	59,95 ± 7,29
		**NS_P**	80,44 ± 12,18	68,82 ± 3,24	87,92 ± 3,34	95,48 ± 8,17
		**NS_E**	130,91 ± 19,52	116,93 ± 3,50	145,21 ± 0,51	143,94 ± 9,05
		**S_E**	46,97 ± 7,05	41,17 ± 3,30	44,73 ± 1,73	50,59 ± 1,92
		**CIV**	141,50 ± 21,91	117,02 ± 10,51	146,96 ± 10,06	153,54 ± 10,23
	FCR	**N_L**	0,10 ± 0,01	0,10 ± 0,01	0,09 ± 0,00	0,07 ± 0,00
		**N_P**	0,37 ± 0,01	0,30 ± 0,05	0,38 ± 0,02	0,41 ± 0,03
		**NS_P**	0,61 ± 0,01	0,59 ± 0,02	0,61 ± 0,02	0,66 ± 0,02
		**S_E**	0,36 ± 0,01	0,35 ± 0,02	0,31 ± 0,01	0,35 ± 0,01
		**CIV**	1,08 ± 0,07	1,01 ± 0,10	1,01 ± 0,07	1,07 ± 0,04
**Motorcortex**	O_2 pmol/(s*mg)	**N_L**	15,15 ± 2,05	13,82 ± 1,77	13,00 ± 1,27	10,67 ± 1,23
		**N_P**	74,6 ± 8,19	65,07 ± 3,15	77,27 ± 4,23	73,30 ± 10,97
		**NS_P**	108,10 ± 12,90	99,02 ± 7,91	114,65 ± 2,82	107,98 ± 14,05
		**NS_E**	153,62 ± 18,18	157,17 ± 12,88	173,04 ± 2,28	164,55 ± 18,18
		**S_E**	55,13 ± 6,52	50,83 ± 4,22	52,01 ± 1,26	51,58 ± 4,20
		**CIV**	184,45 ± 23,16	151,00 ± 27,58	183,35 ± 8,09	167,40 ± 22,73
	FCR	**N_L**	0,10 ± 0,00	0,09 ± 0,01	0,08 ± 0,01	0,07 ± 0,01
		**N_P**	0,49 ± 0,01	0,42 ± 0,02	0,45 ± 0,02	0,44 ± 0,02
		**NS_P**	0,70 ± 0,02	0,63 ± 0,01	0,66 ± 0,01	0,65 ± 0,02
		**S_E**	0,36 ± 0,01	0,32 ± 0,01	0,30 ± 0,00	0,32 ± 0,01
		**CIV**	1,20 ± 0,03	0,95 ± 0,14	1,06 ± 0,05	1,02 ± 0,12

Table S2 – Summary of statistics (two-way ANOVA results to main figures 1 and 2)

		state	genotype	age	interaction
**Striatum**	Per wet weight	**N_L**	F (1, 12) = 1.315, P=0.27	F (1, 12) = 1.709, P=0.22	F (1, 12) = 1.115, P=0.31
		**N_P**	F (1, 12) = 0.239, P=0.63	F (1, 12) = 21.441, **P=0.001 *****	F (1, 12) = 0.231, P=0.64
		**NS_P**	F (1, 12) = 0.099, P=0.76	F (1, 12) = 28.645, **P<0.001 *****	F (1, 12) = 1.267, P=0.28
		**NS_E**	F (1, 12) = 0.004, P=0.95	F (1, 12) = 23.010, **P<0.001 *****	F (1, 12) = 1.496, P=0.25
		**S_E**	F (1, 12) = 1.634, P=0.23	F (1, 12) = 7.398, **P=0.02 ***	F (1, 12) = 2.802, P=0.12
		**CIV**	F (1, 12) = 0.015, P=0.90	F (1, 12) = 5.350, **P=0.04 ***	F (1, 12) = 0.439, P=0.52
	Flux Control Ratio (FCR)	**N_L**	F (1, 12) = 2.910, P=0.11	F (1, 12) = 21.825, **P=0.001 *****	F (1, 12) = 0.216, P=0.65
		**N_P**	F (1, 12) = 0.673, P=0.43	F (1, 12) = 8.873, **P=0.012 ***	F (1, 12) = 1.811, P=0.20
		**NS_P**	F (1, 12) = 0.565, P=0.47	F (1, 12) = 9.373, **P=0.01 ****	F (1, 12) = 0.029, P=0.87
		**S_E**	F (1, 12) = 3.681, P=0.08	F (1, 12) = 18,71 **P=0.001 *****	F (1, 12) = 0.3315 P=0.58
		**CIV**	F (1, 12) = 001, P=0.97	F (1, 12) = 0.374, P=0.55	F (1, 12) = 0.029, P=0.87
**Hippocampus**	Per wet weight	**N_L**	F (1, 12) = 3.319, P=0.09	F (1, 12) = 1.551, P=0.24	F (1, 12) = 0.227, P=0.64
		**N_P**	F (1, 12) = 0.493, P=0.50	F (1, 12) = 6.119, **P= 0.03 ***	F (1, 12) = 1.945, P= 188
		**NS_P**	F (1, 12) = 0.069, P=0.80	F (1, 12) = 4.921, **P=0.05 ***	F (1, 12) = 1.554, P=0.24
		**NS_E**	F (1, 12) = .0489, P=0.50	F (1, 12) = 3.588, P=0.08	F (1, 12) = .340 P=0.57
		**S_E**	F (1, 12) =.000, P=0.99	F (1, 12) = 0.766, P=0.40	F (1, 12) = 2.018, P=0.18
		**CIV**	F (1, 12) = 0.402, P=0.54	F (1, 12) = 2.214, P=0.16	F (1, 12) = 1.212, P=0.29
	Flux	**N_L**	F (1, 12) = 1.758, P=0.21	F (1, 12) =10.240, **P=0.008 ****	F (1, 12) = 0.669, P=0.43
		**N_P**	F (1, 12) = 0.419,	F (1, 12) =4.708,	F (1, 12) = 2.833,

			P=0.53	P=0.051	P=0.12
		**NS_P**	F (1, 12) = 0.659, P=0.43	F (1, 12) = 2.839, P=0.12	F (1, 12) = 4.582, P=0.054
		**S_E**	F (1, 12) = 1.339, P=0.27	F (1, 12) = 2.269, P=0.16	F (1, 12) = 2.557, P=0.14
		**CIV**	F (1, 12) = .009, P=0.93	F (1, 12) = .002, P=0.97	F (1, 12) = 0.726, P=0.41
**Motorcortex**	Per wet weight	**N_L**	F (1, 12) = 1.282, P=0.28	F (1, 12) = 2.689, P=0.13	F (1, 12) = 0.097, P=0.76
		**N_P**	F (1, 12) = 0.854, P=0.37	F (1, 12) = 0.548, P=0.47	F (1, 12) = 0.146, P=0.71
		**NS_P**	F (1, 12) = 0.571, P=0.46	F (1, 12) = 0.554, P=0.47	F (1, 12) = 0.013, P=0.91
		**NS_E**	F (1, 12) = 0.029, P=0.87	F (1, 12) = 0.863, P=0.37	F (1, 12) = 0.174, P=0.68
		**S_E**	F (1, 12) = 0.282, P=0.61	F (1, 12) = 0.070, P=0.80	F (1, 12) = 0.188, P=0.67
		**CIV**	F (1, 12) = 1.299, P=0.28	F (1, 12) = 0.125, P=0.73	F (1, 12) = 0.163, P=0.69
	Flux Control Ratio (FCR)	**N_L**	F (1, 12) = 1.705, P=0.22	F (1, 12) = 9.213, **P=0.01 ****	F (1, 12) = 0.040, P=0.84
		**N_P**	F (1, 12) = 3.826, P=0.07	F (1, 12) = 0.234, P=0.64	F (1, 12) = 2.707, P=0.13
		**NS_P**	F (1, 12) = 6.981, **P=0.02 ***	F (1, 12) = 0.375, P=0.55	F (1, 12) = 4.073, P=0.07
		**S_E**	F (1, 12) = 1.024, P=0.33	F (1, 12) = 12.041, **P=0.005 ****	F (1, 12) = 7.059, **P=0.02 ***
		**CIV**	F (1, 12) = 2.201, P=0.16	F (1, 12) = 0.119, P=0.74	F (1, 12) = 1.199, P=0.30

9
